# Complete Genome Sequence of *Micromonospora* Strain L5, a Potential Plant-Growth-Regulating Actinomycete, Originally Isolated from *Casuarina equisetifolia* Root Nodules

**DOI:** 10.1128/genomeA.00759-13

**Published:** 2013-09-26

**Authors:** Ann M. Hirsch, Johana Alvarado, David Bruce, Olga Chertkov, Peter L. De Hoff, John C. Detter, Nancy A. Fujishige, Lynne A. Goodwin, James Han, Shunsheng Han, Natalia Ivanova, Miriam L. Land, Michelle R. Lum, Nima Milani-Nejad, Matt Nolan, Amrita Pati, Sam Pitluck, Stephen S. Tran, Tanja Woyke, Maria Valdés

**Affiliations:** Department of Molecular, Cell and Developmental Biology, UCLA, Los Angeles, California, USAa; Molecular Biology Institute, UCLA, Los Angeles, California, USAb; Joint Genome Institute, Walnut Creek, California, USAc; Los Alamos National Laboratory, Los Alamos, New Mexico, USAd; Oak Ridge National Laboratory, Oak Ridge, Tennessee, USAe; Departamento de Microbiología, Escuela Nacional de Ciencias Biológicas, I.P.N., Plan de Ayala y Carpio, Mexico City, Mexicof

## Abstract

*Micromonospora* species live in diverse environments and exhibit a broad range of functions, including antibiotic production, biocontrol, and degradation of complex polysaccharides. To learn more about these versatile actinomycetes, we sequenced the genome of strain L5, originally isolated from root nodules of an actinorhizal plant growing in Mexico.

## GENOME ANNOUNCEMENT

*Micromonospora* species live in varied environments, including within legume and actinorhizal root nodules (1–4). Very few *Micromonospora* genomes have been fully sequenced, even though many species enhance plant growth (5). Here, we contribute to this effort with the complete genome sequence of *Micromonospora* strain L5, which was isolated from nodules of *Casuarina equisetifolia* trees growing in Mexico (1, 2).

The *Micromonospora* strain L5 genome was sequenced at the Joint Genome Institute (JGI) using a combination of Illumina (6) and 454 technologies (7). An Illumina GAii shotgun library with reads of 868 Mb, a 454 Titanium draft library with an average read length of 510 to 525 bp, and paired-end 454 libraries with average insert sizes of 10 kb and 14 kb were generated for this genome. All general aspects of library construction and sequencing performed at the JGI can be found at http://www.jgi.doe.gov/. Illumina sequencing data were assembled with Velvet (8), and the consensus sequences were shredded into 1.5-kb overlapped fake reads and assembled with the 454 data. Draft assemblies were based on 793.7 Mb of the 454 draft data and all of the 454 paired-end data. The Newbler parameters were -consed, -a 50, -l 350, -g, -m, -ml 20. The initial Newbler assembly contained 181 contigs in 8 scaffolds.

We converted the initial 454 assembly into a Phrap assembly by making fake reads from the consensus, collecting the read pairs in the 454 paired-end library. The Phred/Phrap/Consed software package (High Performance Software, LLC) was used for sequence assembly and quality assessment (9–11) in the following finishing process. Illumina data were used to correct potential base errors and increase consensus quality by using the software Polisher developed at JGI (A. Lapidus, unpublished data). After the shotgun stage, reads were assembled with parallel Phrap (High Performance Software, LLC). Possible misassemblies were corrected with gapResolution (C. Han, unpublished data) or Dupfinisher (12), or by sequencing cloned bridging PCR fragments with subcloning. Gaps between contigs were closed by editing in Consed, by PCR, and by bubble PCR primer walks. A total of 732 additional reactions and 4 shatter libraries were necessary to close gaps and to raise the quality of the finished sequence.

The genome has a size of 6,907,073 bp, 6,332 predicted open reading frames (ORFs), and a GC content of 72.86%. It most likely has a circular chromosome, based on its close relationship to *M. aurantiaca* (13). We found 4,248 known protein and 1,984 hypothetical ORFs, 52 tRNAs, and 2 rRNAs. The largest number of orthologs was shared with *M. aurantiaca* ATCC 27029 (NCBI accession number NC_014391). Although strain L5 was reported to fix nitrogen via numerous physiological tests (14), we could not find bona fide *nif* gene sequences in the L5 genome even with the use of different sets of *nif* primers. Genes were found for synthesis of a broad range of cell-wall-degrading enzymes, a Sec-independent system, the Tat (twin arginine translocation) export pathway, and type II and VII secretion systems.

### Nucleotide sequence accession number.

The complete sequence of *Micromonospora* L5 has been deposited at NCBI GenBank under accession no. CP002399.

## References

[1] Guillén G Valdés M Liao J Hirsch AM 1993 Identificación de actinobacterias aisladas de nódulos de *Casuarina*, por tecnicas tradicionales y moleculares. Rev. Latinoam. Microbiol. 35:195–200

[2] Niner BM Brandt JP Villegas M Marshall CR Hirsch AM Valdés M 1996 Analysis of partial sequences of genes coding for 16S rRNA of actinomycetes isolated from *Casuarina equisetifolia* nodules in Mexico. Appl. Environ. Microbiol. 62:3034–3036 8702297 10.1128/aem.62.8.3034-3036.1996 PMC168091

[3] Trujillo ME Kroppenstedt RM Schumann P Carro L Martínez-Molina E 2006 *Micromonospora coriariae* sp. nov., isolated from root nodules of *Coriaria myrtifolia*. Int. J. Syst. Evol. Microbiol. 56:2381–2385 17012566 10.1099/ijs.0.64449-0

[4] Trujillo ME Kroppenstedt RM Fernández-Molinero C Schumann P Martínez-Molina E 2007 *Micromonospora lupini* sp. nov. and *Micromonospora saelicesensis* sp. nov., isolated from root nodules of *Lupinus angustifolius*. Int. J. Syst. Evol. Microbiol. 57:2799–2804 18048727 10.1099/ijs.0.65192-0

[5] Hirsch AM Valdés M 2010 *Micromonospora*: a versatile microbe not only for biomedicine, but also for biocontrol and the production of biofuels. Soil Biol. Biochem. 42:536–542

[6] Bennett S. 2004 Solexa Ltd. Pharmacogenomics 5:433–438 15165179 10.1517/14622416.5.4.433

[7] Margulies M Egholm M Altman WE Attiya S Bader JS Bemben LA Berka J Braverman MS Chen YJ Chen Z Dewell SB Du L Fierro JM Gomes XV Godwin BC He W Helgesen S Ho CH Irzyk GP Jando SC Alenquer ML Jarvie TP Jirage KB Kim JB Knight JR Lanza JR Leamon JH Lefkowitz SM Lei M Li J Lohman KL Lu H Makhijani VB McDade KE McKenna MP Myers EW Nickerson E Nobile JR Plant R Puc BP Ronan MT Roth GT Sarkis GJ Simons JF Simpson JW Srinivasan M Tartaro KR Tomasz A Vogt KA Volkmer GA Wang SH Wang Y Weiner MP Yu P Begley RF Rothberg JM 2005 Genome sequencing in microfabricated high-density picolitre reactors. Nature 437:376–380 16056220 10.1038/nature03959 PMC1464427

[8] Zerbino DR Birney E 2008 Velvet: algorithms for de novo short read assembly using de Bruijn graphs. Genome Res. 18:821–829 18349386 10.1101/gr.074492.107 PMC2336801

[9] Ewing B Green P 1998 Base-calling of automated sequencer traces using Phred. II. Error probabilities. Genome Res. 8:186–194 9521922

[10] Ewing B Hillier L Wendl MC Green P 1998 Base-calling of automated sequencer traces using Phred. I. Accuracy assessment. Genome Res. 8:175–185 9521921 10.1101/gr.8.3.175

[11] Gordon D Abajian C Green P 1998 Consed: a graphical tool for sequence finishing. Genome Res. 8:195–202 9521923 10.1101/gr.8.3.195

[12] Han C Chain P 2006 Finishing repeat regions automatically with Dupfinisher, p 141–146 *In* Arabnia HR Valafar H (ed), Proceedings of the 2006 International Conference on Bioinformatics and Computational Biology CSREA Press Athens, GA

[13] Kirby R 2011 Chromosome diversity and similarity within the *Actinomycetales*. FEMS Microbiol. Lett. 319:1–10 21320158 10.1111/j.1574-6968.2011.02242.x

[14] Valdés M Pérez NO Estrada-de los Santos P Caballero-Mellado J Peña-Cabriales JJ Normand P Hirsch AM 2005 Non-*Frankia* actinomycetes isolated from surface-sterilized roots of *Casuarina equisetifolia* fix nitrogen. Appl. Environ. Microbiol. 71:460–466 15640222 10.1128/AEM.71.1.460-466.2005 PMC544234

